# Identification of the ovine mannose receptor and its possible role in Visna/Maedi virus infection

**DOI:** 10.1186/1297-9716-42-28

**Published:** 2011-02-07

**Authors:** Helena Crespo, Ramsés Reina, Idoia Glaria, Hugo Ramírez, Ximena de Andrés, Paula Jáuregui, Lluís Luján, Luisa Martínez-Pomares, Beatriz Amorena, Damián F de Andrés

**Affiliations:** 1 Institute of Agrobiotechnology, CSIC-UPNA-Government of Navarra, Ctra Mutilva, 31192 Mutilva, Spain; 2 Department of Animal Pathology, University of Zaragoza, Miguel Servet 177, 50013 Zaragoza, Spain; 3 School of Molecular Medical Sciences, University of Nottingham, Queen's Medical Centre, Floor A, West Block, Room 1323, Nottingham NG7 2UH, UK; 4 Laboratory of Virology, Genetics and Molecular Biology. FESC, UNAM. C-4, 54700 Cuautitlán Izcalli, State of Mexico, Mexico

## Abstract

This study aims to characterize the mannose receptor (MR) gene in sheep and its role in ovine visna/maedi virus (VMV) infection. The deduced amino acid sequence of ovine MR was compatible with a transmembrane protein having a cysteine-rich ricin-type amino-terminal region, a fibronectin type II repeat, eight tandem C-type lectin carbohydrate-recognition domains (CRD), a transmembrane region, and a cytoplasmic carboxy-terminal tail. The ovine and bovine MR sequences were closer to each other compared to human or swine MR. Concanavalin A (ConA) inhibited VMV productive infection, which was restored by mannan totally in ovine skin fibroblasts (OSF) and partially in blood monocyte-derived macrophages (BMDM), suggesting the involvement of mannosylated residues of the VMV ENV protein in the process. ConA impaired also syncytium formation in OSF transfected with an ENV-encoding pN3-plasmid. MR transcripts were found in two common SRLV targets, BMDM and synovial membrane (GSM) cells, but not in OSF. Viral infection of BMDM and especially GSM cells was inhibited by mannan, strongly suggesting that in these cells the MR is an important route of infection involving VMV Env mannosylated residues. Thus, at least three patterns of viral entry into SRLV-target cells can be proposed, involving mainly MR in GSM cells (target in SRLV-induced arthritis), MR in addition to an alternative route in BMDM (target in SRLV infections), and an alternative route excluding MR in OSF (target in cell culture). Different routes of SRLV infection may thus coexist related to the involvement of MR differential expression.

## Introduction

Visna/maedi virus (VMV) and caprine arthritis encephalitis virus (CAEV) belong to the small ruminant lentivirus (SRLV) group, within the non-oncogenic lentivirus genus of the Retroviridae family, which includes the human immunodeficiency virus (HIV), simian immunodeficiency virus (SIV), feline immunodeficiency virus (FIV), bovine immunodeficiency virus (BIV) and equine infectious anaemia virus (EIAV). Lentiviruses have been classified attending to tropism into those that replicate in macrophages and CD4 T lymphocytes (HIV, FIV, BIV), leading to a decreased T cell number immunodeficiency syndrome, and those that replicate in macrophages but are unable to infect T lymphocytes (SRLV and EIAV). SRLVs infect sheep and goats, and cause, after a variable asymptomatic period, a slow progressive and invariably fatal disease affecting lungs, central nervous system, carpal joints, and/or mammary gland [1,2].

Although different reports have described in vivo infection in cells from mammary epithelium, third eye lid, bone marrow, male reproductive tract, central nervous system and carpal joints, the main target cells for SRLVs in vivo appear to be the monocyte/macrophage lineage. In vitro, viral production has been achieved in a wide spectrum of cell types, synovial membrane and choroid plexus cells being the most commonly used [3,4]. Virus entry has been also detected in cells of heterologous origin such as 293-T human cell line but not in Chinese hamster ovary (CHO) cells even under co-culture conditions, likely due to the absence of a compatible receptor as proposed previously [5]. Like other enveloped viruses, SRLVs enter the host cell by interaction of its envelope (ENV) glycosylated protein (gp135) with cellular receptor(s), allowing the fusion of the virus with the target cell membrane [6]. Studies aimed to identify the SRLV cellular receptor have proposed different candidate molecules, including a 30 kDa membrane-associated proteoglycan substituted with a chondroitin sulphate glycosaminoglycan chain(s) [7]; MHC class II molecules, which incubated with VMV inhibit viral infection, even though infection is not inhibited by class II-specific antibodies [8]; CD4 and CXCR4 molecules, which have been proposed as optional auxiliary components of a VMV receptor (or receptor complex) that facilitate VMV-mediated membrane fusion events [9]; and a complex formed by three membrane proteins of 15, 30 and 50 kDa identified as a Visna binding protein [10]. Nevertheless, none of these molecules has been established as the main essential receptor, an issue which has stimulated research on alternative candidates.

HIV infection of CD4 T cells has been impaired by C-type lectins (such as Concanavalin A, ConA), whose binding to carbohydrate molecules prevents cell fusion and viral entry in culture due to the blockade of ENV [11]. Other lectins anchored to the cell membrane (C-type), such as the mannose receptor (MR) family [12], have an affinity for the glycosylated moieties present in the surface of many pathogens. The MR is present in cells such as monocyte/macrophages, endothelial cells, perivascular microglia, kidney mesangial cells [13,14], tracheal smooth muscle cells [15], Langerhans cells [16] and retinal pigment epithelium [17].

As described in humans, mice, swine and cattle, [18-20], MR is a 180-kDa transmembrane protein with five regions: a cysteine rich ricin-type amino-terminal region, a fibronectin type II repeat, eight tandem C-type lectin carbohydrate-recognition domains (CRD), a transmembrane region, and a cytoplasmic carboxy-terminal tail. The CRD domains, and more specifically CRD4 and CRD5 are essential to recognize mannose, fucose and N-acetyl glucosamine residues. MR recognizes the surface of pathogens and is involved in phagocytosis [14] and endocytosis, mediating antigen processing and presentation, cell migration, intracellular signalling, and pro-inflammatory and anti-inflammatory cytokine production [21]. This receptor is able to bind bacteria, yeast, parasites, and viruses, and links innate to acquired immunity [22]. Blocking the macrophage MR with ligands such as mannan or D-mannose leads to a decreased HIV entry [23]. This study determines the role of mannose-specific lectins on VMV infection and syncytium formation and identifies the ovine MR and its role as an alternative SRLV receptor.

## Materials and methods

### Viruses and cells

Primary cultures of ovine skin fibroblasts (OSF), ovine choroid plexus (OCP) cells, goat synovial membrane (GSM) cells, and goat choroid plexus (GCP) cells, were obtained from SRLV-seronegative animals (tested by ELITEST, Hyphen Biomed; Neuville-Sur-Oise, France) and grown in DMEM medium supplemented with 10% foetal bovine serum and 1% antibiotics/antimycotics mix (Invitrogen, Barcelona, Spain). Chinese hamster ovary (CHO) cells, known to be non-permissive to SRLV infection [5], were grown in F-Ham 12 medium supplemented with 10% foetal bovine serum (Invitrogen). CHO cells permanently transfected with murine MR (kindly provided by Dr Luisa Martinez-Pomares, University of Nottingham, UK) [24] were maintained in F-Ham 12 medium with geneticin (0.5 mg/mL) and also used in infection assays. Blood monocyte-derived macrophages (BMDM) from SRLV-free sheep were obtained by culturing peripheral blood mononuclear cells (PBMC) for 9-days in RPMI 1640 containing GlutaMAX™ I and 25 mM HEPES (Invitrogen) supplemented with 10% foetal lamb serum (Invitrogen), 10 mM sodium pyruvate (Invitrogen), 1% non-essential amino acids (Sigma, Steinheim, Germany), 1% vitamins (Sigma), 50 μM 2-mercaptoetanol (Sigma), and granulocyte macrophage colony-stimulating factor (GM-CSF; kindly provided by Dr Gary Entrican, Moredun Institute, UK) at a concentration of 10 ng/mL.

The EV1 strain [25] was used for in vitro VMV infection assays of BMDM, OSF and GSM cells. Strains EV1, 496 [26] and the infectious clone Kv1772 [27] were used for in vitro infection of CHO and CHO-MR cells. All the infections were performed using 0.1 TCID_50_/cell.

### Amplification, cloning and sequencing of the ovine mannose receptor

RNA was extracted from bronchoalveolar lavage cells of a sheep with SRLV-induced interstitial pneumonia (146/07), using TRIzol reagent (Invitrogen). The DNAse I treated RNA was retrotranscribed to cDNA with SuperScript II (Invitrogen), using oligo-dT as primers and following the manufacturer's instructions. The cDNA obtained was employed to perform overlapping polymerase chain reactions (PCRs). Primers were designed on the highest homology regions among GenBank MR sequences from human, swine and cattle origins. The primer sequences and the amplicon lengths are shown in Table 1. PCR was done using 600 nM final primer concentration and an annealing temperature of 52°C. For retrotranscription and RNA quality control, another PCR was carried out using specific primers for the amplification of a 106 nt fragment of the constitutively expressed β-actin gene [28]. Following agarose gel electrophoresis of PCR products, DNA was purified using Gel/PCR extraction kit (ATP Biotech, Banciao, Taiwan) and cloned into p-GEM-T Easy Vector System (Promega Biotech Iberica, Madrid, Spain). The cloned product was employed to transform electrocompetent *Escherichia coli *XL1-Blue. Transformed bacteria were grown in LB Amp (100 μg/mL) in the presence of X-Gal (50 mg/mL) and IPTG (50 mg/mL). The plasmid DNA was extracted using the Plasmid Miniprep Kit (ATP Biotech). Once the presence of the inserts in the plasmid was confirmed by digestion with EcoRI and the plasmidic DNA from three clones sequenced (Secugen, Madrid, Spain), sequences were analyzed and assembled using the computer software BioEdit, Chromas and MegaAlign. The deduced amino acid sequence and the predicted protein pattern were obtained with the ExPASy Proteomics Tool [29].

**Table 1:** Oligonucleotide sequences used in the PCRs performed in this study and amplicon length.

PCR	Oligonucleotides 5'- 3'	Amplicon length
MR0	MR0Fw CCATGAGGCTACCCCTGCTCCTGGTT MR1Rv GCGTACCACTTGTTTTCAAACTTG	560 nt
MR1	MR1Fw CCGAATCTCAGATTATGAGTGTTGC MR2Rv CTTGCAGATGTAGCCAAGAGGCC	1253 nt
MR2	MR6Fw GGCAAAGATGGATACTGGGCAG CDR5Rv CATTTGCAAAATTGGGTTCACC	1279 nt
MR3	CDR4Fw GGCGAACCTAATAATTATCAG MR7Rv GTGCATCCAGGCAAAAGCATTAC	1210 nt
MR4	MR10Fw ACAGGTGATCCCTCTGGTGAAAGA MR10Rv CTAGATGRCCRCATGTTCRTTCTG	408 nt
qMR (CDR4-CDR5)	MR Fw TGGCAAATCCAGTTGTTAAGATGTT MR Rv AGAATGTTGAATACTGTGGCGAGTT	91 nt
β-actin (control)	Actin 663 Fw CTCACGGAGCGTGGCTACA Actin 769 Rv GCCATCTCCTGCTCGAAGTC	106 nt
qPCR (p17 EV1)	MVV0262Fw CTCCTTGCAGGCCACAATG MVV0333Rv GCTGCTTGCACTGTCTCGG MVV0284P 6-FAM-TGCCTTATGTGTAGTCAGC-TAMRA	71 nt

### Inhibition of viral infection in ConA treated cultures

To evaluate the effects of ConA on viral infection and production, virus (strain EV1) was first preincubated for 60 min at 37°C either with ConA (50 μg/mL), ConA and mannan (1 mg/mL), mannan or medium alone before addition to the cell preparations. OSF and BMDM in DMEM supplemented with 2% FBS or macrophage medium, respectively, were incubated in duplicated 24-well microplates for 1 h with the treated or untreated virus inoculum. Wells were washed with PBS to remove residual inoculum and medium containing lectin (ConA) and/or inhibitor (mannose-rich mannan) was added to a final volume of 1 mL per well. Cells from one plate were collected 16 h post infection (pi) and DNA extracted in order to quantify proviral load. Supernatants from the second plate were collected at day 7 pi, when cytopathic effect (syncytia) was evident in untreated cell cultures (control). Experiments were done in triplicate and repeated three times.

### Luciferase assay

A luciferase assay was performed to ensure that Concanavalin A did not affect viral basal transcription and, as a consequence, viral production. Briefly, 10^5 ^cells/well in 24-well microplates were transfected at a ratio of 1:8 (μg DNA: μL Lipofectamine) using the following plasmids: pGL4.10 [*luc2*] (Promega) as negative control; pGL4.13 [*luc2*/SV40] (Promega), which contains the SV40 promoter as positive control, and pGL4/U3-KV1772 containing the LTR U3 region. Cells were co-transfected with plasmid pRL-SV40 carrying the SV40 promoter and the *Renilla reniformes *luciferase gene as an endogenous control of transfection and after 4 h cells were treated with ConA, ConA and virus (EV1), or medium alone. Following 24 h, cells were lysed with Cell Culture Lysis Reagent 5 × (Promega) and luminescence measured using the Dual-Glo™ Luciferase Assay System (Promega). Luminescence units were normalized to the total protein present in each sample, which was quantified by the Bradford assay (BioRad, Madrid, Spain). Firefly luciferase activity was normalized to renilla luciferase activity. Results were expressed as luciferase units/ng of the total protein of each sample. Each experiment was done in triplicate and was repeated three times.

### Inhibition of syncitium formation by Concanavalin A in *env*-transfected cultures

To test whether glycosylated ENV mediates cell fusion allowing syncytium formation in the absence of virus, ovine skin fibroblasts (OSF) were transfected with pN3-*env *plasmid (pN3 with VMV *env *gene encoding the precursor protein gp150 [30]), using Lipofectamine (Invitrogen) at a ratio of 1:6 (DNA: Lipofectamine 2 mg/mL) and following manufacturer's instructions. ENV-containing transformants were selected in the presence of geneticin (0.5 mg/mL of medium). Empty plasmid (pN3) was used as negative control. To determine the possible ConA-mediated inhibitory effect, ConA (50 μg/mL) was added 5, 24 and 48 h following transfection. Syncytium formation was evaluated by optical microscopy up to 72 h pi, after Giemsa staining. Experiments were repeated twice.

### Blocking of MR by mannan

Different concentrations of mannan (4, 2, 1, 0.5, 0.25 and 0.125 mg/mL) were administered to cultured OSF (negative control), GSM cells and blood monocyte-derived macrophages (BMDM, 10^5 ^cells/well in two 24-well microplates). Following incubation (30 min at 37°C), the virus (Ev1) was added (0.1 TCID_50_/cell) and cells were cultured for 16 h. After washing in PBS, one plate was used for DNA extraction and provirus quantification (q-PCR). For RT activity determinations, the second plate was further incubated until day 7, when syncytia appeared in untreated cells. These blocking experiments were repeated three times.

### Real time PCRs

A real time PCR technique (q-PCR) was used to determine proviral DNA for quantifying viral entry-integration upon ConA and mannan treatments 16 h pi. Briefly, OSF, GSM cells and BMDM (10^5 ^cells/well) from the ConA and/or mannan experiments (see below) were washed and fresh medium was added. Cells were harvested and DNA extracted with Qiamp DNA Blood Mini Kit (Qiagen, Hilden, Germany), according to the manufacturer's protocol. GAG (p17) segments were quantified by q-PCR as described previously [28] using ovine DNA from VMV infected cultures and oligonucleotides reported in Table 1. Results were expressed as provirus copy number/ng DNA. Tests were carried out in duplicate and repeated three times.

Expression of MR was quantified using β-actin as a housekeeping gene by substracting the corresponding ΔCt value from that obtained in MR using cDNA from BMDM, GSM, OSF, CHO or CHO-MR cultured cells. Specific primers for amplification of the ovine β-actin and the CDR4-CDR5 region of ovine MR are shown in Table 1.

### Reverse Transcriptase (RT) activity assay

RT activity was measured in cell culture supernatants according to the manufacturer's instructions (HS-Lenti RT Activity kit, Cavidi, Uppsala, Sweden) as an indicator of productive infection. The signal intensity thus obtained was used to produce a standard curve in order to quantify the virus, using as reference standard serial dilutions of the same viral strain titrated by the classical Reed-Muench method [31]. Experiments were done in duplicate and repeated at least twice.

### Western blot and ICC

OSF, CHO, CHO-MR and GSM cell lysates (40 μg) were used in Western blot. A rabbit anti-human MR polyclonal undiluted serum (cat. No. ab64693 Abcam, Cambridge, UK) and two mouse anti-human MR monoclonal antibody reagents (clones 8 and 15. Personal communication, Luisa Martinez-Pomares, University of Nottingham, UK) were used as primary antibodies at a dilution of 1/100. Anti-rabbit or anti-mouse (Thermo Scientific, Erembodegem, Belgium) peroxidase-labelled secondary antibodies were employed accordingly at a dilution of 1/2000. The reaction was developed using Supersignal West Pico Chemiluminiscent substrate (Thermo Scientific). The same cells were also used following standard ICC protocols. Briefly, cells were washed in PBS and fixed in methanol:acetone (1:1) for 5 min. In the case of BMDM, an additional peroxidase blocking step was carried out using 5% H_2_O_2 _in methanol for 5 min. Following washing, cells were blocked for 1 h using 2.5% casein and 5% lamb serum. Primary antibody was added undiluted (in the case of monoclonal antibodies) or diluted in PBS containing 1.25% casein. After washing, anti-rabbit or anti-mouse peroxidase-labelled secondary antibodies were added at a 1/2000 dilution. The reaction was developed with diaminobenzidine (DAB).

### Statistical analysis

The normal distribution of the data was confirmed by Shapiro-Wilks and Kolmogorov-Smirnov tests. The absorbance values between different treatments were compared by Student's *t*-test for related samples. Data obtained from real time PCR were analysed by Wilcoxon non-parametric test for related samples.

### GenBank accession number

The assembled complete ovine MR sequence was submitted to GenBank and given accession number HM099914.

## Results

### Characterization of the ovine mannose receptor (MR) encoding sequence and comparison with other MRs

Taking into account the relevance of interaction of glycosylated ENV with the soluble C-type lectin ConA on VMV infection and cell fusion, we attempted the identification of the gene encoding a cell surface-anchored C-type lectin, the ovine MR, to determine subsequently its possible role in VMV infection. Nucleotide sequence analysis revealed that the complete ovine MR had a similarity of 86 and 89% with the complete human and porcine MR, respectively. *Bos taurus *MR concatenated sequence from GenBank, which included the amino-terminal cysteine-rich domain, the fibronectin type-II domain and seven of the eight known MR CDRs, revealed the highest overall similarity (91%) with the ovine counterpart. When comparing the similarity between MR domains considered individually, CDR1, CDR2, CDR4 and CRD5 were the most highly conserved regions of MR across species (human, swine and ovine; Table 2). The deduced amino acid sequence of the ovine MR presented the same structure as that of MR from humans, swine or cattle: an extracellular region containing an amino-terminal cysteine-rich domain, a fibronectin type-II domain, eight CRDs, a transmembrane region and a short cytoplasmic tail. Both the ovine CDR4 and CDR5 domains conserved a characteristic WND motif, which has been reported to be a feature of the C-type lectins [18]. The ovine sequence also conserved the FENTLY and the di-aromatic Y-F motifs in the cytoplasmic domain, FENTLY being relevant to the endocytic receptor internalisation and di-aromatic Y-F motif being involved in the endosomal sorting signal [32]. The predicted amino acid sequence of ovine MR had an identity of 87% and 90% with MR of humans and swine, respectively.

**Table 2:** Similarity values (%) between ovine MR predicted amino-acid sequence with that from human, swine and bovine species.

Domain of ovine MR	Species		
	
	Human	Swine	Bovine
Cystein rich domain	93.33	95.55	96.66
FN-II	91.67	90.00	96.66
CRD1	91.60	93.13	99.23
CRD2	90.48	93.88	98.63
CRD3	79.86	91.37	98.02
CRD4	94.74	96.05	99.28
CRD5	90.28	90.97	NA
CRD6	82.23	89.17	97.45
CRD7	78.95	83.46	93.98
CRD8	88.81	91.61	97.90
TM + cytoplasmic tail	76.77	80.81	96.96

FN-II: fibronectin type-II domain; CRD: carbohydrate recognition-like domain; TM: transmembrane region. NA: sequence not available.

### Presence of ovine MR transcripts

The presence of ovine MR transcripts was investigated using MR CDR4-CDR5-specific RT-PCR in a panel of cell types, including ovine GSM cells, BMDM and OSF as well as CHO and CHO-MR cells, using β-actin as endogenous expression control to confirm RNA quality. PCR amplification and sequencing revealed that MR transcripts were only found in GSM cells and BMDM (Table 3). Since the ovine MR encoding sequence structure is compatible with a cell surface anchored protein, as it occurs in other mammalian species, detection of the ovine MR protein was attempted by WB and ICC using three reagents (two monoclonal antibodies and a polyclonal serum) to human MR. CHO cells transfected with mouse MR were used as positive control. ICC results always yielded unspecific perinuclear staining of cells from ovine origin whereas in WB, monoclonal antibodies to human MR failed to detect mouse MR (likely due to the lack of cross-reactivity), whereas the polyclonal serum against human MR detected only murine MR on the CHO-MR cell surface which expression has been previously shown [24]. Unfortunately, none of the three reagents cross-reacted with the ovine MR expressed on GSM cells or BMDM. Thus, ovine MR-specific antibody inhibition experiments analogous to those carried out with mannan could not be done, because antibodies were unavailable.

**Table 3:** Relative MR transcripts expression in different cell types.

	**Ct MR**	**sd**	**Ct β-actin**	**sd**	**ΔCt**	**100 × 2**^**-ΔCt**^
	
BMDM	31.78	± 0.08	28.58	± 1.72	3.21	10.841646
GSM	36.16	± 0.06	28.59	± 0.04	7.57	0.526263
OSF	45.00	ND	18.46	± 0.13	ND	ND
CHO	45.00	ND	17.78	± 1.71	ND	ND
CHO-MR	31.32	± 0.02	16.79	± 0.64	14.53	0.004227

### Effect of the soluble lectin Concanavalin A on VMV infection and syncytium formation

**Figure 1:**
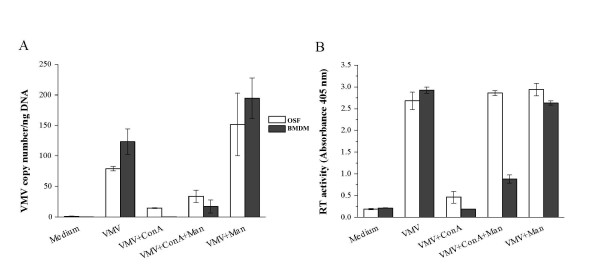
**Concanavalin A (ConA) mediated inhibition of visna/maedi (VMV) infection**. Virus was preincubated with ConA, ConA-mannan or mannan alone for 1 h and then used to infect ovine skin fibroblasts (OSF) and blood monocyte derived macrophages (BMDM). Inhibitory effects of these treatments were measured by proviral load quantification (VMV copies per ng of DNA; A); and RT activity (absorbance at 405 nm) in culture supernatants 7 days after inoculation (B). Values are the mean ± SE of the assays performed.

ConA is a cell-free lectin with a high capability of binding mannosylated residues able to inhibit HIV infection. Since non-glycosylated VMV ENV does not allow cell fusion, we determined if ConA could inhibit VMV infection and syncytium formation, and if this inhibition diminished or disappeared upon addition of mannan. For this purpose, the virus (VMV Ev1) was preincubated with ConA and added to OSF and BMDM cultures. Proviral load, determined 16 h after inoculation by q-PCR, was decreased upon addition of VMV-ConA in OSF and BMDM (*P *< 0.05; Figure 1A). Mannan partially, but significantly, restored proviral load in cultures of OSF and BMDM inoculated with ConA treated virus (*P *< 0.05; Figure 1A). There was no RT activity in 16 h culture supernatants (not shown), but 7 days pi, a highly significant (*P *< 0.01) reduction of RT activity in both OSF and BMDM (Figure 1B) infected with ConA treated virus was observed. Preincubation of virus with ConA and mannan completely restored RT activity in OSF culture supernatants (to values similar to those obtained in OSF with untreated virus), and partially restored this activity in BMDM culture supernatants. Preincubation of virus with mannan alone did not cause any effect on RT activity.

**Figure 2:**
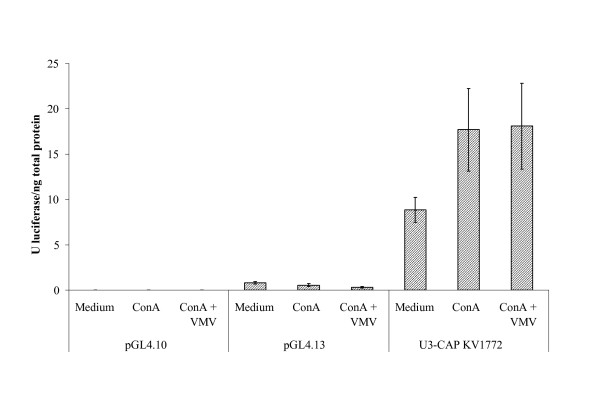
**LTR promoter activity in ovine skin fibroblasts (OSF)**. OSF were transfected with pGL4/U3-KV1772 (clone containing VMV LTR), pGL4.13 containing SV40 promoter (as positive control), or pGL4 for basal activity (as negative control). Transfected cells were treated with ConA, ConA and VMV or medium and the LTR activity was measured 24 h pi using the Luciferase reporter system (Promega). Firefly luciferase activity was normalized to renilla luciferase activity. Data are expressed as luciferase units/ng total protein of each sample. Values are the mean ± SE of assays performed.

To exclude the possibility that ConA itself resulted in decreased VMV LTR transcriptional activity (rather than inhibiting viral entry), either ConA alone or ConA and VMV (at the same concentrations as those used in experiments corresponding to Figure 1) was added to OSF cells transfected with the pGL4/U3-KV1772 plasmid, which contained a LTR site driving a luciferase gene reporter system. In this system, ConA either alone or combined with VMV did not cause any inhibitory effect on LTR transcriptional activity (*P *> 0.05; Figure 2). Altogether, these results showed that ConA inhibited VMV provirus integration into the cell genome as well as productive infection, and that this inhibitory effect was at least partially counteracted with mannan.

Next, we determined if ConA affected syncytium formation through its interaction with the VMV-ENV glycoprotein. For this, cells (OSF, easy to transfect) were transfected with an ENV-encoding pN3-plasmid [30]. After 5 h, syncytia started to appear and cultures showed thereafter an extended syncytium formation to the end of the experimental period (72 h pi). Addition of ConA to pN3-*env *transfected cultures after the 5-h time point did not have any effect on the subsequent appearance of syncytia. However, treatment with ConA within 5 h after transfection inhibited syncytium formation 24, 48 or 72 h pi (Table 4). Thus, ConA impaired syncytium formation, most likely by blocking mannosylated residues of ENV.

**Table 4:** Evaluation of syncytium formation by optical microscopy in pN3-Env and pN3 transfected OSF cells following Concanavalin A addition at different time points.

		Syncytium formation evaluation		
		24 h	48 h	72 h
		
	ConA addition time points (h)			
	5	-	-	-
pN3-ENV transfected OSF	24	+	++	++++
	48	+	++	++++

	5	-	-	-
pN3 transfected OSF	24	-	-	-
	48	-	-	-

### Mannan blocking of VMV infection

Under the hypothesis that GSM cells and BMDM (and not OSF) express MR on the cell membrane, we assessed the role of MR in VMV infection using GSM cells, BMDM, and OSF by a mannan-mediated blocking approach.

**Figure 3:**
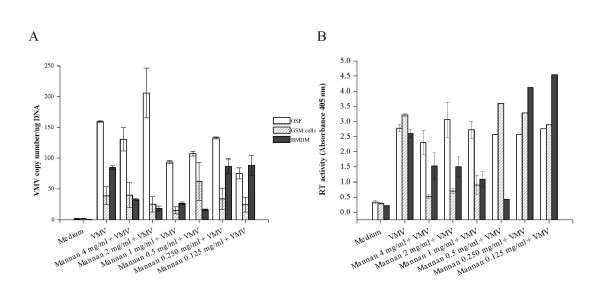
**Effect of mannan on VMV infection**. Cell cultures of OSF, GSM cells and BMDM were pre-treated with mannan at different concentrations for 30 min. Proviral load from DNA (A) and RT activity in the supernatant (B) of these cultures were determined 16 h and 7 days pi respectively. Values are the mean ± SE of assays performed.

The effect of mannan on VMV infection was studied by determining proviral load in the three culture systems GSM cells, BMDM and OSF (Figure 3A) 16 h pi. The addition of mannan resulted in a decreased proviral copy number (about two fold) in GSM cells and BMDM (*P *< 0.05), but it had no effect in OSF (*P *> 0.05).

Results compatible with these observations were obtained when studying viral production (RT activity in culture supernatants) 7 days pi. About 4 and 2.5 fold reduction in RT activity was observed in GSM cells (*P *< 0.0001) and BMDM (*P *< 0.05), respectively (Figure 3B). The effect was dependent on mannan concentration, decreasing beyond 2 mg/mL. Finally, the addition of mannan to OSF cultures, even at high concentrations (1-4 mg/mL), did not alter the production of virus (*P *> 0.05), as these cells were lacking MR transcripts, which strongly suggests lack of MR protein.

Altogether, these results indicate that the ovine MR was expressed in at least two cell types (GSM cells and BMDM) susceptible to VMV infection, but not in OSF.

### VMV infection via heterologous MR

**Figure 4:**
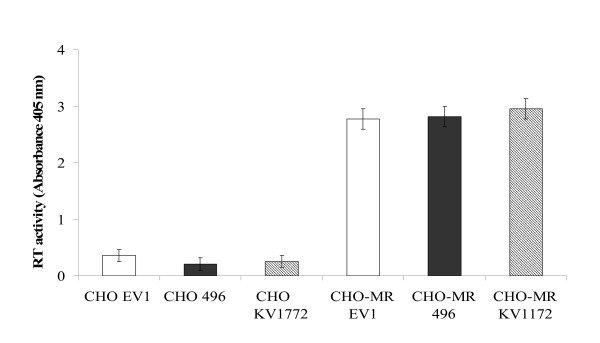
**SRLV infection of CHO cells expressing murine MR**. RT activity of CHO and CHO-MR cell cultures infected with the strains EV1, 496 and KV1772 was determined (Absorbance 405 nm) in the supernatants 7 days pi. Values are the mean ± SE of assays performed.

In the absence of an ovine MR cell expression system, CHO-MR (cells permanently expressing mouse MR according to WB), and CHO cells (not expressing MR) were infected with the VMV strains Ev1 [25], 496 [26] and the infectious clone Kv1772 [27]. Uninfected CHO and CHO-MR cells were used as control. VMV proviral load was measured by q-PCR 16 h pi reaching 80.7 ± 9 copies/ng of DNA in the case of CHO-MR cells infected with Ev1. After seven days of culture, RT activity in the supernatants of all the CHO-MR cultures yielded positive values (Figure 4). Controls remained negative in both tests. Syncytium formation was confirmed with Giemsa staining in all cases. This culture supernatant was also used to inoculate OSF cultures. Remarkably, no RT activity was found 12, 21, 33 and 47 days pi, suggesting that although CHO cells were permissive and expressed heterologous (mouse) MR, they did not support VMV productive infection.

## Discussion

The role of innate immunity is gaining interest in the field of lentiviral infections [33,34]. An important component of innate immunity is the MR (CD206), a group VI C-type lectin, present at the cell surface and endosomes [14]. This study identifies the ovine MR genetic characteristics, the corresponding nucleotide and deduced amino acid sequences and the putative protein structure, all of which were found very close to those found in other mammals [35,36]. Expression of the ovine MR is being attempted at present through transfection of SRLV non-permissive cells.

In the host, differences in MR oligomerization [37], heterogeneity of MR N-glycosylation [38], as well as variation in individual genetic makeup [39] and health status [40] may account for differences in MR expression. Like in other species, the ovine MR expression differed among cell types, mRNA specific transcripts being present in macrophages (BMDM) and synovial membrane (GSM) cells, but not in skin fibroblasts (OSF). In SRLV pathogenesis, GSM cells are known to be infected in vivo [41] and if these cells do differentially express MR on the cell surface in vivo as they do in vitro, MR could represent an entry pathway of ENV-mannosylated viruses through carpal joint infection.

The polyclonal reagent available to detect MR protein expression was produced against a 57-amino acid peptide of the human MR, but the limited size of this peptide (about 3.9% of the whole MR protein) and sequence differences (12 mismatches) between this human MR peptide and the homologous peptide of ovine origin may have led to the evident lack of cross-reactivity of the polyclonal reagent with the ovine MR. In contrast, this polyclonal antibody reacted with mouse MR (with 16 mismatches in the peptide region compared to human MR). The kind and site of amino acid substitutions may account for the differences in cross-reactivity of the polyconal reagent to the ovine vs. the mice peptide.

Evidence of VMV infection in CHO cells transfected with MR (from species like mice) supports the hypothesis that MR expression is sufficient in vitro for VMV infection in particular cell types. In a previous work [5] CHO/mouse somatic cell hybrid lines became permissive to SRLV if they included mouse chromosome 2 or 4. The finding that mouse chromosome 2 contains the MR gene, originally named *Mcr *in that species [42], may explain this finding and suggests that the involvement of the membrane associated C-type lectin DC-SIGN (dendritic cell-specific ICAM-grabbing non-integrin) in this permissiveness (and not that of MR) could be excluded, since DC-SIGN is encoded by chromosome 8 (and not chromosomes 2 or 4). Similarly in our study, the involvement of DC-SIGN was unlikely, since dendritic cells were not used. However, redundant or alternative pathways of virus capture via lectins may coexist [43].

The observation that CHO cells become infected, upon transfection with MR from species not susceptible to VMV (mice), may indicate a non species-specific viral interaction with the MR. However, the CHO-MR cells infection by VMV was not productive, indicating that subsequently to viral integration, factors involved in viral production appear to differ when comparing CHO-MR with GSM cells (expressing MR), the latter being capable of productive infection.

There must be routes of virus entry into skin fibroblasts (OSF) other than mannose binding lectins, as in these cells blockade of infection by mannan did not take place but a productive VMV infection was observed. Accordingly, OSF appeared to bind, via a yet unidentified receptor different from MR, ENV viral protein glycosylated residues, taking into account the need of ENV glycosylated residues for cell fusion [38], and the observation that viral replication was strongly inhibited in these cells by ConA and restored upon addition of mannan (likely by mannan binding to ConA). This ConA-mediated inhibition of virus entry and syncytium formation is in agreement with results in human cells using different carbohydrate-binding agents and HIV-1 strains [44].

The macrophages (BMDM) used in this study represent another category of cells, which express MR transcripts but appear to produce at least two types of VMV receptors, MR and an additional unknown receptor. Mannan, when added to the virus preparation, abrogated ConA effects but only partially in these cells, according to RT activity and provirus quantification. Furthermore, when mannan was added to the cells, it inhibited infection (as it occurred in GSM cells), but only partially. MR has been proposed in different species as a main virus binding site in particular cells [45] (this might apply to GSM cells used in this study) and/or an auxiliary molecule in other cells (this would apply to BMDM employed in this work). In non-phagocytic cells, MR is not acting as a professional phagocytic receptor, since it does not lead to clearance of virus [46]. Accordingly, in our study the VMV entry into GSM cells via MR subsequently led to a productive infection. However in human cells such as macrophages, results on significance of MR-mediated HIV-1 endocytosis are inconclusive [45]. This may be due in part to the fact that lentiviruses such as HIV-1 bind cells at least via two independent pathways that may coexist in macrophages, the currently accepted infectious route by plasma membrane protein receptors and the route mediated by the endocytic MR, through which HIV-1 epitopes may be subjected to exogenous MHC class I presentation (cross-presentation) [23]. If this applies to the VMV model, the second route would not be present in skin fibroblasts (OSF), as they lacked MR transcripts and were not susceptible to mannan-mediated blocking, but would exist in GSM cells and BMDM.

However, caution should be taken when studying MR expression in BMDM, as it may vary along the individual's life, having implications in pathogenesis. Based on the macrophage phenotype classification in subclinical vs. clinical stages [38] and according to this and our previous studies [28], macrophages of SRLV asymptomatic sheep (such as the BMDM tested in this study) would exhibit increased B7 transcript production, whereas those of clinically affected sheep would be expected to have an increased MR expression and viral infection. The known downregulation of B7 molecule expression [28] and a Th2-biased antibody response to the viral infection [47] occurring in VMV clinical disease, including arthritis [26], would be compatible with an upregulation of MR expression in particular target organs such as carpal joints. Antibodies against MR are currently being developed for immunohistochemical studies at different stages of VMV infection.

Besides the cellular receptor, the genetic makeup of the virus may determine the virus-cell interactions, since the number and distribution of ENV amino acids susceptible to mannosylation, may affect viral entry through membrane lectins and consequently the viral production and appearance of disease. Further studies on ENV composition and viral entry are warranted.

In conclusion, we report in vitro studies demonstrating that concerning viral entry there are at least three main patterns in target cells capable of generating a productive infection: i) particular cell types such as synovial membrane (GSM) cells may use MR as a VMV main infection route; ii) other cells such as fibroblasts (OSF) use a route other than MR to bind the glycosylated ENV allowing the virus entry to the cell; and iii) there are cells like macrophages (BMDM), a classical SRLV target, that use MR and an additional receptor for VMV entry. The three cell types may be used as in vitro models to explore the mechanisms and relative relevance of the different entry routes in VMV infections and provide the basis for studies in vivo, on tropism, viral receptors and MR expression aimed to understand viral pathogenesis and host progression from asymptomatic to clinical stages.

## Competing interests

The authors declare that they have no competing interests.

## Authors' contributions

HC and RR participated in the conception and design of this study and the drafting of the manuscript. HC also carried out the molecular genetics and virological studies. IG, HR, XA and PJ participated on PCR, virus preparation and titration. LL provided bronchoalveolar lavage samples and participated in the designing and writing of the manuscript. LMP provided MR antibodies and helped in writing of the manuscript. BA and DA participated in the experimental design, search for funding resources, work supervision and the focus and discussion of the manuscript. All authors read and approved the final manuscript.
